# Early Castration in Horses Does Not Impact Osteoarticular Metabolism

**DOI:** 10.3390/ijms242316778

**Published:** 2023-11-26

**Authors:** Marion Rouge, Florence Legendre, Razan Elkhatib, Christelle Delalande, Juliette Cognié, Fabrice Reigner, Philippe Barrière, Stefan Deleuze, Vincent Hanoux, Philippe Galéra, Hélène Bouraïma-Lelong

**Affiliations:** 1 Université de Caen-Normandie, OeReCa, 14000 Caen, France; marion.rouge@unicaen.fr (M.R.); razankhatib@hotmail.com (R.E.); christelle.delalande@unicaen.fr (C.D.); vincent.hanoux@unicaen.fr (V.H.); 2 Université de Caen Normandie BIOTARGEN, 14000 Caen, France; florence.legendre@unicaen.fr (F.L.); philippe.galera@unicaen.fr (P.G.); 3 INRAE, Université de Tours, Centre de Recherche de Tours, UMR PRC, 37380 Nouzilly, France; juliette.cognie@inrae.fr; 4 INRAE, Université de Tours, Centre de Recherche de Tours, UEPAO, 37380 Nouzilly, France; fabrice.reigner@inrae.fr (F.R.); philippe.barriere@inrae.fr (P.B.); 5 Université de Liège, 4000 Liège, Belgium; s.deleuze@uliege.be

**Keywords:** horse, castration, steroid hormones, cartilage, bone, in vivo metabolism

## Abstract

The castration of stallions is traditionally performed after puberty, at around the age of 2 years old. No studies have focused on the effects of early castration on osteoarticular metabolism. Thus, we aimed to compare early castration (3 days after birth) with traditional castration (18 months of age) in horses. Testosterone and estradiol levels were monitored from birth to 33 months in both groups. We quantified the levels of biomarkers of cartilage and bone anabolism (CPII and N-MID) and catabolism (CTX-I and CTX-II), as well as of osteoarthritis (HA and COMP) and inflammation (IL-6 and PGE_2_). We observed a lack of parallelism between testosterone and estradiol synthesis after birth and during puberty in both groups. The extra-gonadal synthesis of steroids was observed around the 28-month mark, regardless of the castration age. We found the expression of estrogen receptor (ESR1) in cartilage and bone, whereas androgen receptor (AR) expression appeared to be restricted to bone. Nevertheless, with respect to osteoarticular metabolism, steroid hormone deprivation resulting from early castration had no discernable impact on the levels of biomarkers related to bone and cartilage metabolism, nor on those associated with OA and inflammation. Consequently, our research demonstrated that early castration does not disrupt bone and cartilage homeostasis.

## 1. Introduction

Steroid hormones, such as testosterone and estradiol, are derived from cholesterol during steroidogenesis. Testosterone serves as a direct precursor to estradiol and is converted by an enzymatic complex composed of cytochrome P450 and aromatase, which is encoded by a single gene, *Cyp19a1* [1,2]. Two distinct postnatal periods of testosterone synthesis have been observed in males. Specifically, a transient peak in synthesis is observed during the perinatal period [3]. Subsequently, testosterone decreases significantly, followed by a subsequent increase to peak levels during puberty [4]. In adulthood, these levels remain high until a decline in elderly men. In contrast to testosterone, the synthesis of estradiol is characterized by a single increase in production during puberty. Following this period, estradiol levels remain low in men. Estradiol synthesis is carried out by various tissues in men, with the testis estimated to be responsible for 20% of the total synthesis [5].

The endocrine function of the testis, along with the widespread distribution of steroid hormone receptors in various tissues, highlights the range of physiological processes regulated by steroid hormones [6]. Numerous studies conducted in humans and rodents have shown that testosterone and estradiol play a significant role in the physiology of both bone and cartilage [7,8,9,10]. The biological effects of these two steroids are mediated through their specific receptors: the classical estrogen receptors ESR1 and ESR2, and the androgen receptor AR. Specifically, ESR1, ESR2, and AR have been demonstrated to be expressed in osteocytes, osteoblasts, and osteoclasts for bone, as well as in chondrocytes for cartilage [11,12,13,14,15]. 

Testosterone and estradiol play essential roles in the growth, development, and maintenance of the skeleton in various species [16,17]. During the pubertal stage, the elevation of steroid hormones is associated with the peak in bone growth observed during this period [18,19]. Moreover, sex steroid hormones contribute significantly to skeletal sexual dimorphism [15,20,21]. In addition, testosterone plays a crucial role in growth and contributes to periosteal apposition, which is radial growth. These differences result in longer bones (8%) and greater bone width, as well as 25% more bone mass in men compared to women [22]. Puberty concludes with the closure of the epiphyseal growth plate. Indeed, at the culmination of puberty, the elevated concentrations of estrogens stimulate the closure of the epiphyses through their influence on growth plate chondrocytes, a process occurring in both boys and girls [18].

The pivotal role of steroid hormones in maintaining bone homeostasis has been demonstrated in clinical instances of gonadal failure [23,24], cases involving *ESR1* gene mutations [25,26] or *CYP19A1* gene mutations [27], and in mouse models [28,29,30,31]. It has also been established that estrogens play a considerably more significant role in human bone metabolism than testosterone [32]. Steroid hormones also contribute to maintaining articular cartilage homeostasis [8,10,33]. 

In horses, castration is a common surgical procedure performed to mitigate the fiery and sometimes aggressive behavior of stallions. This practice involves suppressing the primary production of gonadal steroids, in particular testosterone, which influences behavior through its impact on the brain [34]. The stallion is the male mammal that produces the highest levels of testicular estrogens, distinguishing it from other species [35,36]. Currently, castration is typically performed after puberty, during the phase of skeletal growth (usually before the age of 2) or later, around the age of 3, once growth has concluded, with the goal of attaining the morphological characteristics of the stallion [37,38]. Performing this procedure at an earlier stage is uncommon, and limited information is available regarding its potential consequences. 

In our previous study, the impact of early-age castration in neonates (at 3 days of age) compared to traditional castration (at 18 months of age) on the physical and behavioral development of horses was evaluated [39]. We found no pre-, intra-, or post-operative complications related to early castration in foals. Moreover, early castration did not disrupt the morphological or behavioral development of horses, as observed in individuals followed up to the age of 3 years. In this context, the objective of the present study is to assess the medium-term side effects of early castration compared to traditional castration on osteoarticular metabolism, with a focus on the circulating levels of testosterone and estradiol in the same cohort of horses. First, we investigated the protein expression of steroid receptors (AR and ESR1) in bone and cartilage tissues to determine if these tissues are targets for steroids. We also examined the presence of enzymes responsible for the final step in the synthesis of testosterone (CYP17A1) and estradiol (CYP19) in neonatal testes to assess the contribution of the testes to the circulating levels of these hormones. Additionally, we explored the impact of early and traditional castration on bone anabolism and catabolism. To accomplish this, we quantified biomarkers indicating bone (cross-linked C-telopeptide of type I collagen or CTX-I) and cartilage (cross-linked C-telopeptide of type II collagen or CTX-II), catabolism, biomarkers of bone (N-terminal midfragment of osteocalcin or N-MID) and cartilage (carboxypropetide of type II collagen or CPII) anabolism, and biomarkers of OA (hyaluronic acid or HA as well as cartilage oligomeric matrix protein or COMP) and inflammation (prostaglandin E2 or PGE_2_ and interleukin 6 or IL-6) in serum or plasma from birth to 33 months of age for both groups of horses. 

## 2. Results

### 2.1. Investigation of ESR1 and AR Detection in Horse Bone and Cartilage Tissues

**Figure 1:**
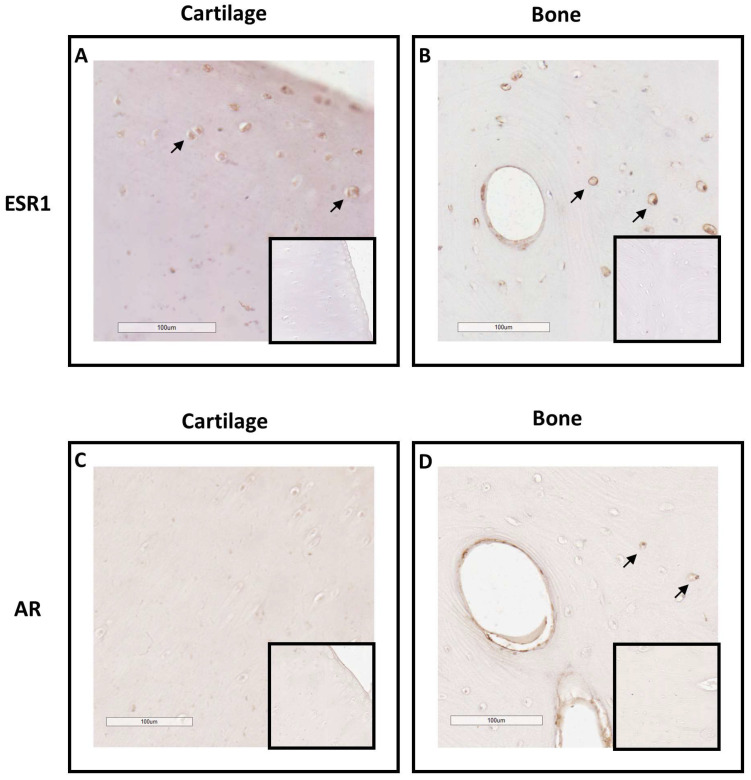
Immunolocalization of ESR1 and AR in cartilage (**A**,**C**) and bone from post-pubertal horses (**B**,**D**). Sections from cartilage and bone exhibited immunostaining for ESR1 in chondrocytes (arrow in (**A**)) and in osteocytes (arrow in (**B)**). Sections from cartilage and bone showed immunostaining for AR in a few osteocytes (arrow in (**D**)), but not in chondrocytes (**C**). The insert did not display immunostaining for ESR1 and AR when the section was incubated with the secondary antibody only. The images shown are representative of the experimental results obtained from three horses (100 μm scale bar).

To explore the equine skeleton as a gonadal steroid target, the protein expression of ESR1 and AR was examined in the cartilage and bone tissues of post-pubertal horses using immunohistochemistry (Figure 1). ESR1 protein was mainly detected in the nucleus of chondrocytes of the hyaline cartilage (Figure 1A), as well as in the nucleus and on the plasma membrane of osteocytes and around the outskirts of the Haversian canal inside the osteon of cortical bone (Figure 1B). In comparison, no ESR1 staining was detected in the cells of the control sections of cartilage and bone. As for AR, no chondrocytes expressed AR (Figure 1C). A small number of osteocytes within the osteon exhibited the expression of this receptor. The expression of AR was also observed at the periphery of the Haversian canal within the osteon and in its concentric lamellae (Figure 1D). These findings suggest that estradiol could influence both bone and cartilage metabolism, while testosterone may only impact bone metabolism since AR was exclusively located within bone structures.

### 2.2. Analysis of Circulating Levels and Testicular Synthesis of Testosterone in Both Study Groups 

**Figure 2:**
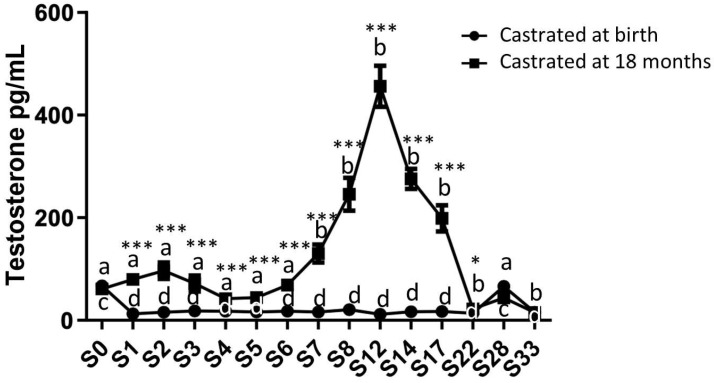
Circulating levels of testosterone in plasma samples from horses castrated at birth and at 18 months. Plasma testosterone levels were measured for 33 months after birth in the two experimental groups using ELISA (S0 to S12: n = 11, S14 to S17: n = 8, and S28 to S33: n = 5). Data are presented as mean ± SEM. A variation analysis in testosterone levels compared to P0 within the same group was conducted using a Kruskal–Wallis test, followed by a Dunn’s comparison (a ≠ b; c ≠ d). A Mann–Whitney test was performed to compare the testosterone levels at the same time between the two groups (* *p* ≤ 0.05; *** *p* ≤ 0.001).

Circulating testosterone levels were measured from birth up to 33 months using plasma samples collected from both study groups (Figure 2). As expected, there was a fluctuation in group 1 (the horses castrated at 18 months) between 0 and 33 months. Indeed, an initial increase in the circulating levels of testosterone was observed during the first months after birth, peaking at around 2 months. Subsequently, a second significant increase in plasma testosterone levels occurred between 7 and 17 months, reaching its maximum synthesis at 12 months (456.1 pg/mL ± 40.02). After castration at 18 months, a decline in circulating testosterone levels was observed, eventually reaching levels slightly above those in horses castrated at birth (22 months: 22.37 pg/mL ± 3.43). 

In group 2 (the horses castrated at birth), the circulating testosterone level was consistently low but remained detectable, registering as early as 1 month (12.85 pg/mL ± 1.33), in contrast to the control group, and it remained stable until 22 months. Subsequently, in both groups, an increase in testosterone levels was observed at 28 months, even after castration, reaching levels comparable to those measured at birth (43.66 pg/mL ± 6.58 for group 1 and 66.37 pg/mL ± 8.66 for group 2). 

**Figure 3:**
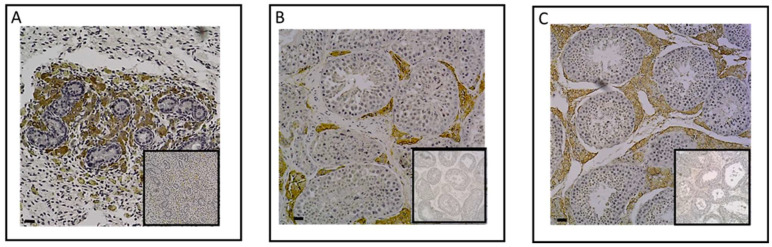
Immunodetection of CYP17A1 protein in neonatal (3 days) (n = 8) (**A**), post-pubertal (18 months) (n = 8) (**B**), and adult (n = 6) (**C**) testes. A positive CYP17A1 signal was observed in the neonatal testicular interstitial tissue with 10x magnification (**A**). Post-pubertal and adult testes were used as positive controls (**B**,**C**). The insert did not show immunostaining for CYP17A1 when the section was incubated with the secondary antibody only. Images shown are representative of the experimental results (100 μm scale bar).

To determine whether plasma testosterone levels were associated with gonadal synthesis in neonates, we examined the protein expression of the enzyme CYP17A1, which plays a role in the final step of androgen synthesis. Through an immunohistological analysis, we successfully identified the expression of this enzyme in the testes of neonates (Figure 3A). Specifically, CYP17A1 was immunolocalized within Leydig cells of the interstitial tissue of the testes in 3-day-old foals, as observed in post-pubertal and adult testes (Figure 3B,C), confirming the testis’ capacity to synthesize androgens throughout the perinatal period.

### 2.3. Analysis of Circulating Levels and Testicular Synthesis of Estradiol in Both Study Groups 

**Figure 4:**
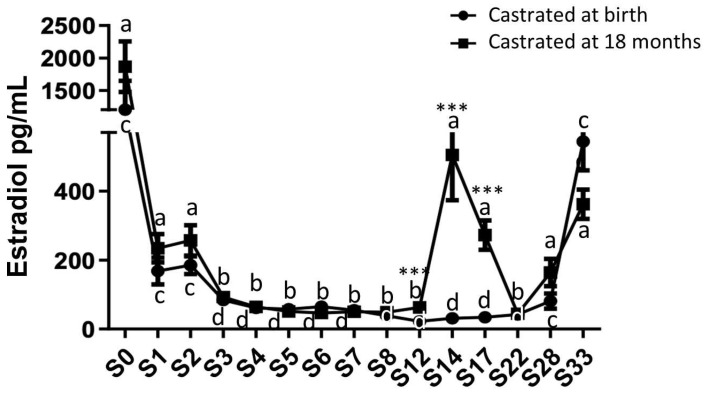
Circulating levels of estradiol in plasma samples from horses castrated at birth and at 18 months. Plasma estradiol levels were measured over 33 months after birth in the two experimental groups using ELISA (S0 to S12: n = 11, S14 to S17: n = 8, and S28 to S33: n = 5). Data are presented as mean ± SEM. A variation analysis in estradiol levels compared to P0 within the same group was conducted with a Kruskal–Wallis test, followed by a Dunn’s comparison (a ≠ b; c ≠ d). A Mann–Whitney test was performed to compare estradiol levels at the same time between the two groups *** *p* ≤ 0.001).

Estradiol levels were quantified in plasma samples collected from both study groups from birth up to 33 months (Figure 4). 

High estradiol levels were observed at birth in both study groups (1866.75 pg/mL ± 390.96 for group 1, castrated at 18 months and 1202.6 pg/mL ± 445.5 for group 2, castrated at birth). A substantial reduction (approximately 8.5-fold) in plasma estradiol levels was observed in both groups starting from 1 month after birth, with no significant difference between the two groups (group 1: 211.17 pg/mL ± 35.78 and group 2: 146 pg/mL ± 33.80). Following this significant decline, these levels gradually decreased for the next 3 months and remained consistently low in both groups until 12 months (about 56 pg/mL). Subsequently, after reaching one year of age, a significant increase in estradiol levels was observed in group 1, peaking at 14 months (409.80 pg/mL ± 109.67). After the castration of horses at 18 months (group 1), the reduction in estradiol levels continued, reaching levels equivalent to those of horses castrated at birth by 22 months (group 2: 44.48 pg/mL ± 12.04). In both study groups, estradiol levels increased after 22 months, regardless of early or late castration, reaching levels by 33 months similar to those observed during the pubertal peak at 14 months (362 pg/mL ± 42.49 for group 1 at 33 months and 544.04 pg/mL ± 84.07 for group 2). These findings indicate the extragonadal synthesis of estradiol.

**Figure 5:**
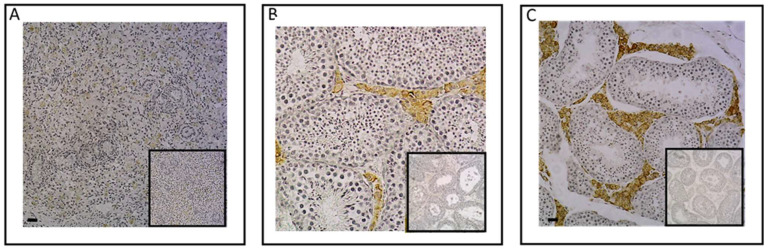
Immunodetection of aromatase in neonatal (3 days) (n = 8) (**A**), post-pubertal (18 months) (n = 8) (**B**), and adult (n = 6) (**C**) testes. No positive aromatase signal was observed in the neonatal testicular interstitial tissue at 10x magnification (**A**). Post-pubertal and adult testes were used as positive controls (**B**,**C**). The insert did not show immunostaining for aromatase when the section was incubated with the secondary antibody only. Images shown are representative of the experimental results (100 μm scale bar).

To determine whether plasma estradiol levels at birth could be linked to gonadal synthesis, the presence of the aromatase enzyme, responsible for the aromatization of testosterone into estradiol, was examined in the testes of 3-day-old animals (Figure 5A). However, the immunohistological analysis did not reveal the presence of aromatase in the testes of 3-day-old foals, unlike post-pubertals and adults in whom aromatase is strongly expressed in the Leydig cells of the interstitial tissue (Figure 5B,C).

### 2.4. Analysis of Circulating Levels of Osteoarticular Metabolism Biomarkers in Both Study Groups 

**Figure 6:**
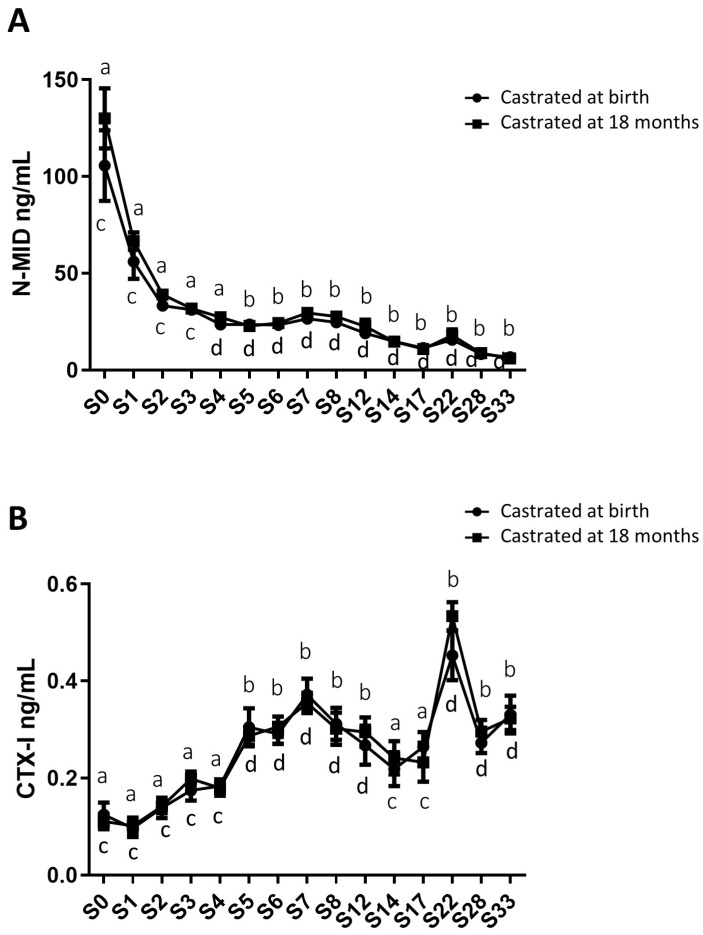
Circulating levels of biomarkers of bone metabolism in blood samples from horses castrated at birth and at 18 months. Plasma N-MID levels (**A**) as well as serum CTX-I levels (**B**) were measured for 33 months after birth in the two experimental groups using ELISA (S0 to S12: n = 11, S14 to S17: n = 8, and S28 to S33: n = 5). Data are presented as mean ± SEM. A variation analysis in the biomarker levels compared to P0 within the same group was performed using a Kruskal–Wallis test followed by a Dunn’s comparison (a ≠ b; c ≠ d). A Mann–Whitney test was conducted to compare the biomarker levels at the same time between the two groups, and no differences were observed.

**Figure 7:**
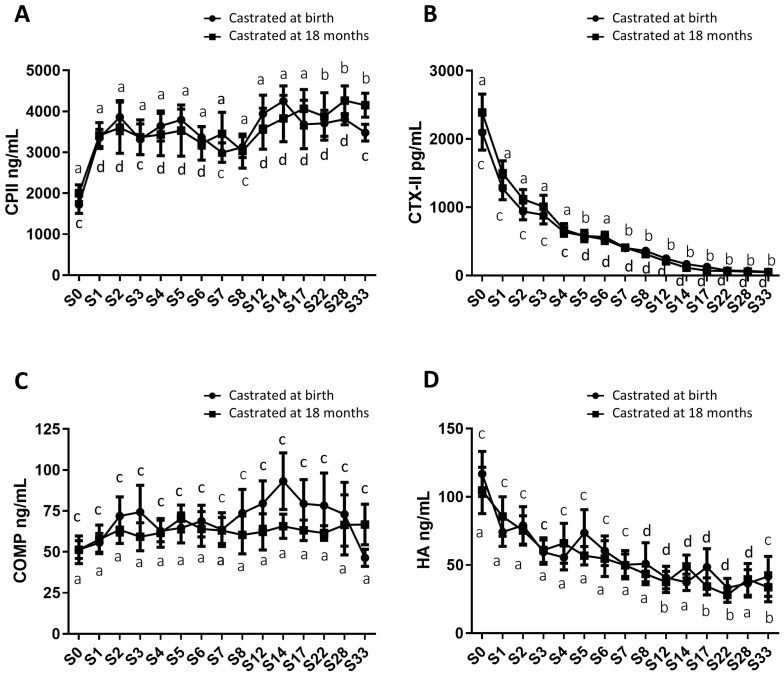
Circulating levels of cartilage and osteoarthritis biomarkers in serum samples from horses castrated at birth and at 18 months. Serum levels of CPII (**A**), CTX-II (**B**), COMP (**C**), and HA (**D**) were measured for 33 months after birth in the two experimental groups using ELISA (S0 to S12: n = 11, S14 to S17: n = 8, S28 to S33: n = 5). Data are presented as mean ± SEM. A variation analysis in the biomarker levels compared to P0 within the same group was performed with a Kruskal–Wallis test followed by a Dunn’s comparison (a ≠ b; c ≠ d). A Mann–Whitney test was compared to compare the biomarker levels at the same time between the two groups, and no differences were observed.

In addition to gonadal biomarkers, aspects of the osteo-articular status were studied to assess the impact of sex steroid deprivation on osteoarticular metabolism. Thus, biomarkers of bone anabolism (N-MID; Figure 6A) and catabolism (CTX-I; Figure 6B), cartilage anabolism (CPII; Figure 7A) and catabolism (CTX-II; Figure 7B), and osteoarthritis (COMP and HA; Figure 7C,D) were quantified from birth to 33 months using blood samples collected from both groups of castrated horses. The profiles of these biomarkers were consistent between both groups regardless of the castration stage, with no statistically significant differences. Thus, the levels of different biomarkers were not affected by castration. However, their levels displayed fluctuations over time. 

As for bone markers, N-MID levels gradually decreased over time in a similar manner for both study groups, declining from an average level of 117.9 ng/mL at birth to an average level at 6.3 ng/mL at 33 months for the entire group, indicating an 18.7-fold decrease (Figure 6A). In contrast, CTX-I levels progressively increased until 33 months (going from an average level of 0.118 ng/mL at birth to an average level of 0.327 ng/mL at 33 months for the entire group), representing a 2.7-fold increase (Figure 6B), with no significant differences observed between the two groups. We observed a peak in CTX-I levels at 22 months, with an average level of 0.493 ng/mL. These findings indicate an enhancement of bone matrix catabolism during foal growth, regardless of whether castration was performed early or late. These results may also suggest an increase in the turnover of the bone ECM (an imbalance between osteoformation/osteolysis) during foal growth.

For cartilage markers, CPII levels progressively increased approximately two-fold from birth to 33 months, and this trend was consistent in both study groups, with average levels of 1862.5 ng/mL at birth and 3815.9 ng/mL at 33 months for the entire group (Figure 7A). The CTX-II levels continuously and significantly decreased about 44-fold over the same period, and this decline occurred similarly in both study groups, with average levels of 2243.4 ng/mL and 51.3 ng/mL at birth and at 33 months for the entire group (Figure 7B). These findings support the notion of cartilage anabolism during foal growth, regardless of whether castration was performed early or later.

Furthermore, serum markers of osteoarthritis, including COMP and HA, were also evaluated. COMP levels remained stable and comparable between both study groups throughout the analysis, with average levels of 62.2 ng/mL for group 1 (castrated at 18 months) and 69.1 ng/mL for group 2 (castrated at birth) (Figure 7C). HA levels exhibited a gradual decrease in both study groups from birth to 33 months, with average levels of 110.7 ng/mL at birth and 37.6 ng/mL at 33 months for the entire group, representing approximately a three-fold decrease. This reduction showed no significant difference between the two groups (Figure 7D). 

Moreover, pro-inflammatory biomarkers such as IL-6 (Supplementary Material Figure S1A) and PGE_2_ (Supplementary Material Figure S1B) were measured in both study groups. These markers are produced in osteoarticular pathologies such as arthritis, and to a lesser extent, OA. Elevated levels of these markers were detected at birth, reflecting the animal’s inflammatory state following parturition. The levels of these two markers similarly decreased after birth in both study groups, and their levels did not seem to be influenced by the surgical procedure of castration. There was an observed progressive decrease in IL-6 levels (approximately a 2.8-fold decrease from birth to 33 months for the entire group), while PGE_2_ levels exhibited a significant decrease during the first 2 months of the animal’s life (approximately a 3.8-fold decrease for the entire group), followed by a gradual decline until 33 months (approximately a 5.5-fold decrease from two months to 33 months, with average levels of 80.6 pg/mL at 33 months for the entire group). Overall, these findings suggested that neither the act of castration nor the timing of castration affected the levels of osteoarthritis and inflammatory biomarkers.

## 3. Discussion

In equines, castration is a common breeding practice. However, it is performed at various ages due to the lack of consensus among veterinarians and breeders. Castration is rarely performed in the early stages, and its effects on osteoarticular development have not been thoroughly investigated. It is proposed that castration might be one of the mechanisms contributing to osteoarticular pathologies [10,23,40], potentially affecting animal welfare and increasing costs for breeders. However, unlike other farmed species, such as cattle and pigs, which are commonly castrated to enhance farming industry performance before their slaughter, horses are intended for a prolonged career. This study is part of a project that aims to compare the medium-term effects of equine castration at 3 days of age (early castration, before the pubertal action of steroids) with castration at 18 months of age (traditional castration, after the pubertal action of steroids). We have already conducted two comparative studies on the surgical procedure of early and late castration in horses. These studies have shown that early castration does not impact physical and behavioral development, and they also demonstrate the non-contribution of the equine testes to vitamin D metabolism [39,41]. In this study, we evaluated the osteoarticular metabolism’s side effects resulting from early castration in comparison to traditional castration performed up to the age of 33 months. 

Firstly, we confirmed the perinatal synthesis of testicular androgens by the immunodetection of the CYP17A1 enzyme in the testes of 3-day-old horses. Our study unveiled a lack of parallelism between testosterone and estradiol plasma levels both post-birth and during puberty in horses. Testosterone synthesis in stallions is characterized by an initial, transient perinatal elevation, a phenomenon described in various species and referred to as ‘mini-puberty’ [3,42,43]. The absence of steroid exposure during mini-puberty in group 2 horses castrated at birth could potentially impact animal behavior. Indeed, it has been noted that mini-puberty allows for the masculinization and defeminization of the central nervous system in males [44,45]. This developmental period involves changes in total brain volume, cortex thickness, and cortical network development [46,47,48]. As a result, early castration might influence the behavior of horses due to the absence of mini-puberty. Potential psychological or behavioral effects of early castration could also indirectly impact physical development. However, our previous study within the same cohort demonstrated no differences between early or traditional castration in terms of temperament and behavior at either 1 or 3 years of age, and also in terms of physical development [39]. The assessed temperament traits included the mother–foal bond, reactivity to humans, sensory sensitivity, gregariousness, fearfulness towards novelty and suddenness, and activity. The variables measured regarding weight and morphometric monitoring were body weight, height at withers, body length, chest perimeter, chest width, hip width, distal limb length, and metacarpal width [39]. We demonstrated that early castration does not interfere with morphological and behavioral development, suggesting no impact of early castration on osteoarticular metabolism, in accordance with our present results. This study evaluated the impact of castration in the medium term; we cannot exclude the possibility that long-term effects could manifest. 

In contrast, estrogen synthesis in stallions was characterized by high estradiol levels at birth, which markedly decreased during the first months of life. These elevated post-birth estradiol levels likely originated from the mother. Indeed, it has been described that within the 48h preceding parturition, 17β-estradiol levels are twice as high in mares [49]. This hypothesis was confirmed by the absence of the CYP19A1 protein, as demonstrated through immunohistochemistry in the testes of 3-day-old animals. Subsequently, estradiol levels increased at the onset of puberty; however, this pubertal estradiol peak was both delayed and more transient (spanning from 14 to 17 months) than the pubertal testosterone peak. This delayed occurrence of the estradiol peak could potentially be attributed to different regulations of steroidogenesis enzymes in equine testes.

The monitoring of plasma steroid levels (testosterone and estradiol) revealed that horses castrated at birth (group 2) are not exposed to mini-puberty or the pubertal peak in testosterone and estradiol, unlike horses subjected to traditional castration at 18 months (group 1). Furthermore, even after castration at birth, measurable levels of steroids persist. The circulating testosterone levels in castrated horses exhibited another dynamic alteration following early or late orchidectomies. Notably, a slight and transient testosterone peak was observed at 28 months in both groups. Subsequently, the testosterone levels returned to the baseline. In the present study, we focused on testosterone and estradiol as factors influencing osteoarticular metabolism. However, other factors secreted by the testes are essential to regulate osteoarticular metabolism, in particular vitamin D. In a prior study, we examined the impact of both early and traditional castration on vitamin D levels [41]. No changes in bioactive vitamin D3 levels were observed after castration regardless of the age at the time of surgery: 1 week, after puberty (2 years), or in adulthood (6 years), suggesting no impact of vitamin D3 on osteoarticular metabolism.

The temporal fluctuations in steroid levels following castration reveal a significant synthesis of androgens by extra-gonadal tissues. However, a question arises regarding the origin of the steroids detected after an orchidectomy. The activity of the CYP17A1 enzyme, which is involved in androgen synthesis, has been reported to be abundant in the testes, with low levels detected in the adrenal glands [50,51]. While the testes are responsible for 95% of circulating levels of testosterone in males, the adrenal cortex has been shown to have the ability to synthesize androgens like dehydroepiandrosterone (DHEA). DHEA can subsequently be converted into testosterone and, consequently, into estradiol in tissues that express various steroidogenic enzymes responsible for this synthesis, including bones [15,52]. Moreover, in humans, DHEA, dehydroepiandrosterone sulfate (DHEAS), and androstenedione are primarily secreted by the zona reticularis of the adrenal cortex [53]. These hormones play a crucial role as the primary substrate source for the extragonadal synthesis of sex steroids. In humans, it has been observed that the secretion of adrenal androgens increases during adrenarche in children, peaking between the ages of 20 and 30 [54,55]. If we extend this timeframe to horses, it could potentially correspond to the peak in testosterone levels observed in 28-month-old horses. Adrenal androgens might play a role in complementing testicular androgens after castration in horses, as androstenedione has been suggested to enhance bone accretion during growth [56]. Moreover, low levels of DHEA have been linked to a higher incidence of osteoporosis [57]. In addition to the aromatization of adrenal androgens, estrogens can also originate from adipose tissue. Indeed, a study conducted on ovariectomized rats demonstrated an increase in extragonadal aromatization by adipose and liver tissues, as well as by the adrenal glands, resulting in elevated blood estradiol levels [58]. The increase in estradiol levels occurring around 28 months, regardless of the age of castration, could be attributed to variations in body composition during the horse’s growth period. It has indeed been noted that the proportion of adipose tissue increases between the ages of 12 and 30 months in horses [59]. Adipose tissue has been identified as capable of synthetizing certain steroids like estrogens [60]. Therefore, this tissue could potentially serve as the source of the elevated estradiol levels documented in our study. Other tissues expressing aromatase (skin, bone, and brain) [60] could also produce estrogens in the absence of gonadal sources.

Regarding osteoarticular metabolism, we have demonstrated that the depravation of steroid hormones due to early castration does not affect the levels of biomarkers associated with bone and cartilage metabolism (no differences were observed in the levels of N-MID, CTX-I, CPII, and CTX-II between both study groups). This suggests that castration has no impact on bone loss and cartilage homeostasis. This unique result contradicts the findings observed in humans [7,23,24,61,62] and rodents [28,29,63], as well as in studies related to cartilage [8,10,64,65]. Nevertheless, this is consistent with our previous results which demonstrated a low or negligible contribution of the testes to vitamin D bioactivation in horses, whereas vitamin D plays a crucial role in maintaining bone health [41]. It is possible that the low levels of steroids quantified after early castration might still be sufficient to exert their effects on bone and cartilage tissues. The absence of protein expression of AR in cartilaginous tissue and the lower expression of AR in bone tissue compared to that of ESR1 support the idea that estrogens play a much more significant role than androgens in the regulation of bone and cartilage metabolism. These data align with findings from studies conducted in human or in rodent model species of gonadal failure or steroid hormone receptor mutations where steroid synthesis is disrupted [25,26,30]. For instance, hypogonadal men with inadequate testicular steroid hormone synthesis [23,24,27] experience a loss of bone mass and an increased risk of fractures. Additionally, hormonal imbalances have also been linked to bone disruption [62]. Indeed, an excess of estrogen leading to an increase in bone mass density (BMD) was demonstrated in male mice overexpressing aromatase [30]. Steroid hormones also play a role in cartilage tissue [10,66]. A difference in the prevalence of OA between men and women has been well established. In women, OA rarely appears before the age of 45, but its frequency increases considerably after menopause, suggesting that estrogen deficiency could be a factor in the disease’s onset [8,10]. 

Among the bone markers investigated, N-MID is a specific marker of bone formation, while CTX-I is used as a marker of bone resorption, aiding in the assessment of osteoporosis risk [67,68]. We observed that N-MID levels exhibited a gradual decrease, while CTX-I levels increased from birth to 33 months, regardless of early or late castration. These findings suggest a potential bone loss, which contrasts with the typical bone growth observed in foals. Another explanation we can propose is that these data may also reflect an increase in turnover and metabolic activity in the ECM of bone tissue—in other words, an enhancement in the osteolysis/osteoformation balance. Moreover, it is important to consider that these data need to be correlated with a juvenile population. Indeed, serum levels of bone turnover markers are not stable across the lifespan and tend to be higher in infants and children compared to adults [69,70]. Unlike adults, children have elevated concentrations of bone markers due to their rapid skeletal growth and heightened bone turnover rate [71]. As a result, complementary measurements in adult horses indicated that the levels of these two bone markers at 33 months tend to align with adult. Nevertheless, we did not identify any differences between the two study groups. It might have been informative to complement these results with bone densitometry data and the measurements of other bone markers, such as alkaline phosphatase, for example. 

In terms of cartilage markers, CP-II is a specific marker of cartilage anabolism, and CTX-II is a specific marker for cartilage degradation [72,73]. Our observations indicate that CPII levels experience a gradual and consistent increase, while CTX-II levels exhibit a continuous and significant decrease from birth to 33 months, regardless of the castrated horses’ group. These findings support the notion of cartilage anabolism during foal growth, regardless of early or late castration. This may be correlated with the development of the growth plate in foals. Despite cartilage degradation observed in OA being primarily correlated to aging, to our knowledge, no study has yet correlated the levels of cartilage markers to OA in a pediatric population. To address this gap, we expanded our analyses by measuring COMP and HA levels in both groups of horses. Indeed, serum COMP levels have a strong correlation with the degree of OA [72,74], and serum HA levels may serve as a valuable predictor of OA progression [75,76]. We demonstrated that COMP levels remained consistent, and HA levels gradually decreased in both study groups from birth to 33 months, regardless of the age of castration. Additionally, pro-inflammatory biomarkers (IL-6 and PGE_2_) were not affected by the age of castration. Taken together, these findings imply that neither castration nor the timing of castration impacted cartilage homeostasis and fate.

## 4. Materials and Methods

### 4.1. Animals and Sample Collection

The animal requirements adhered to the European Community Council Directive 2010/63/UE. 

This study focused on two groups of 22 male Welsh ponies from an experimental herd within the PAO experimental unit (UEPAO; INRAE Val de Loire, Nouzilly, France). All experimental procedures received approval from the Val de Loire Ethic committee (authorization N° APAFlS#4530-20l603l4150l1475 v3) [39,41]. Foals were born in three consecutive seasons as follows: cohort 1 of 10 foals, cohort 2 of 6 foals, and cohort 3 of 6 foals. These animals were categorized based on their age at the time of castration:

- GROUP 1: Traditional castration: 11 foals were castrated at 18 months (5 from cohort 1, 3 from cohort 2, and 3 from cohort 3, respectively). Horses in this group were castrated at 18 months and served as the control group for the other experimental group.
- GROUP 2: Early castration: 11 neonates were castrated at birth (5 from cohort 1, 3 from cohort 2, and 3 from cohort 3, respectively). Horses underwent early castration 3 days after birth.

Blood samples (S) were collected concurrently with morphometric measurements from birth until 33 months: S0 to S12, n = 11 (from all 3 cohorts), S14 to S17, n = 8 (from the first 2 cohorts), and S28 to S33, n = 5 (from the first cohort only). 

The blood samples were collected in either dry tubes or tubes containing anticoagulants (Heparin or EDTA) to obtain serum or plasma, respectively. Subsequently, the tubes were centrifuged at 3000 r.p.m. for 10 min at 4 °C to separate the supernatants, which were then stored at −80 °C until ELISA was performed.

The testicular tissues, obtained after castration of horses from the two groups of Welsh ponies, were derived from the middle of the testis. Testes from adult horses (with an average age of 6.6 years) were collected during routine castrations at the Saint-Michel du Livet veterinary clinic in Calvados, France to serve as a positive control for immunohistological analysis. Testes were fixed in 4% PFA and embedded in paraffin for subsequent histological analysis. 

Osteochondral sections were harvested from the medial part of the distal aspect of the metacarpal condyle of French Standardbred horses (median age of 3 years) as part of another study [77]. The study protocol received approval from the ComEth Anses/ENVA/UPEC Ethical Committee (permit number: 10/03/15-12). Osteochondral sections from joints treated with a physiological saline placebo were fixed with a formalin solution (Sigma-Aldrich, Saint-Louis, MI, USA), decalcified (RDO, Eurobio Scientific, Courtaboeuf, France), and embedded in paraffin for subsequent histological analysis. 

### 4.2. Biomarker Assays

Testicular, osteoarticular, and inflammation biomarker levels were measured using ELISA kits following the manufacturer’s instructions, with either plasma or serum samples. Each sample was quantified in duplicate. The kit references used in this study, along with their respective detection limits, are detailed in Table 1. 

### 4.3. Immunohistochemistry

#### 4.3.1. Testicular Tissue

Immunohistochemistry was performed on 5 μm testicular sections mounted on glass slides that had been deparaffinized and rehydrated, following a previously described procedure [41]. To inhibit endogenous peroxidase activity, all sections were subjected to 10 min of incubation in a 3% H_2_O_2_/PBS solution. Subsequently, antigen retrieval was accomplished by immersing the sections in a sodium citrate buffer (0,1M; pH 6) and heating them in a microwave oven at 90W for 10 min. After the heating step, the sections were allowed to cool for 40 min and then rinsed with PBS. To permeabilize the sections, the glass slides holding them were treated with a 0.2% Triton/PBS solution for 20 min. Afterward, the testicular sections were blocked with PBS containing 3% BSA for 20 min and subsequently incubated with the corresponding primary antibody at 4 °C overnight in a humidified chamber. After three washes in PBS, the sections were exposed to the compatible secondary antibody for 1h30. Immunoreactivity was visualized using an indirect immunoperoxidase method following a brief rinse. A positive signal is identified through the response of the diaminobenzidine chromogen (Sigma-Aldrich, Saint-Louis, MI, USA). Following counterstaining with Mayer’s hematoxylin (BIOCYC GmbH & CO., KG, Potsdam, Germany), the sections were dehydrated and covered with Eukitt mounting medium (ORSAtec GMBH, VWR International SAS, Rosny-sous-Bois, France). Table 2 outlines the primary and secondary antibodies. As a negative control, the primary antibody was omitted.

#### 4.3.2. Osteochondral Tissue

Immunohistochemistry was performed on deparaffinized and rehydrated 5 μm osteochondral sections mounted on glass slides. An additional decalcification step required 10 min of incubation in 9% formic acid (Sigma-Aldrich, Saint-Louis, MI, USA). Subsequently, the bone sections underwent a 30 min retrieval/blocking procedure in a solution containing 0.5% hyaluronidase and 3% BSA (Sigma-Aldrich, Saint-Louis, MI, USA). To facilitate permeabilization, the glass slides holding the sections were exposed to a 0.2% Tween 20/PBS solution for 10 min. Following this, the sections were incubated in a 1.5% H_2_O_2_/PBS solution (Sigma-Aldrich, Saint-Louis, MI, USA) for 20 min. After being rinsed three times in PBS, the sections were placed in a humidified chamber and incubated with the appropriate primary antibody at 4 °C overnight. After three PBS washes, the sections were exposed to a compatible secondary antibody for a duration of 1.5h Following exposure to the secondary antibody, the osteochondral sections were washed in PBS, and immunoreactivity was visualized using the diaminobenzidine detection system (Sigma-Aldrich, Saint-Louis, MI, USA). After counterstaining with Mayer’s hematoxylin (BIOCYC GmbH & CO., KG, Potsdam, Germany), the sections were dehydrated and subsequently covered with Eukitt mounting medium (ORSAtec GMBH, VWR International SAS, Rosny-sous-Bois, France). The antibodies used are summarized in Table 2. As a negative control, the primary antibody was omitted.

### 4.4. Statistical Analyses

All data were reported as mean ± SEM. Statistical analyses were conducted using GraphPad Prism 8.0.1 software (San Diego, CA, USA). A Kruskal–Wallis test, followed by a Dunn’s multiple comparisons test, was performed to compare biomarker concentration levels with P0 within each experimental group (castrated at 18 months and at birth). Additionally, a Mann–Whitney test was used for statistical analysis to compare samples at the same time between the two groups. Differences were considered significant at *p* ≤ 0.05.

## 5. Conclusions

**Figure 8:**
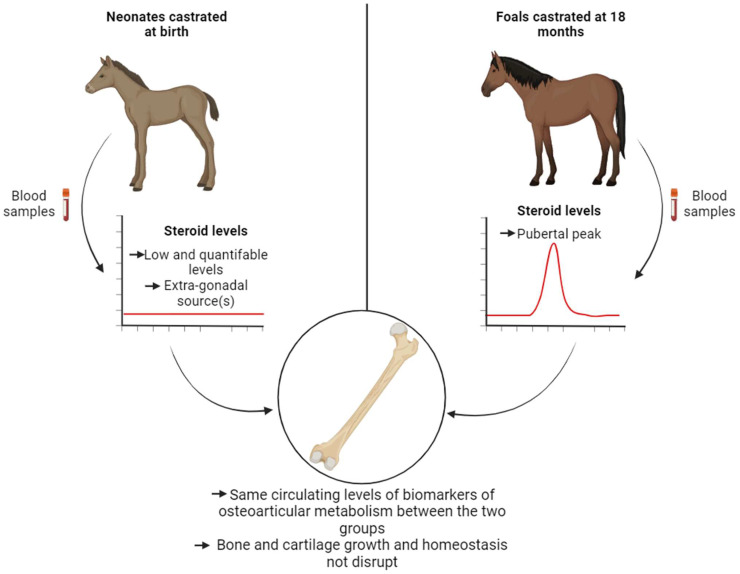
Early castration has no short- and medium-term effects on bone and cartilage growth in foals.

This study produced results indicating that there are no discernable side effects of castration on osteoarticular metabolism in horses, whether the procedure is performed 3 days after birth (early castration) or at 18 months (traditional castration) (Figure 8). Specifically, the removal of the pubertal source of gonadal steroid synthesis did not disrupt the levels of biomarkers associated with bone and cartilage metabolism during the growth and maturation of the skeleton. We have highlighted that extra-gonadal source(s) of steroids are non-negligible and could contribute to maintaining bone and cartilage homeostasis. Further investigations are needed to identify extra-gonadal source(s) of steroids. Consequently, our study complements other investigations associated with this project and reinforces the idea that horses represent an unique model in endocrinology [39,41]. Taken together with our previous studies, we have confirmed that there are no contraindications to early castration. This is supported by the absence of complications related to the surgical procedure, the behavior and morphology of the animals, and their osteoarticular metabolism.

## Figures and Tables

**Table 1:** References for the ELISA kits used.

Biomarker	Reference	Limits of Detection	Type of Sample
**Testosterone** **Sex steroid hormone**	ADI-901-176, Enzo Life Sciences (Lyon, France)	3.9–1000 pg/mL	Plasma
**17β-estradiol** **Sex steroid hormone**	951456, Cayman Chemical, Interchim SA, Montluçon, France	6.6–4000 pg/mL	Plasma
**CTX-I** **Bone catabolism**	AC-02F1, Immunodiagnostic Systems, Dijon, France	0.02 ng/mL	Serum
**N-MID Osteocalcin** **Bone anabolism**	AC-11F1, Immunodiagnostic Systems, Dijon, France	0.5 ng/mL	EDTA plasma
**CPII** **Cartilage anabolism**	60-1003, IBEX pharmaceuticals Inc, Montréal, Canada	Unspecified	Serum
**CTX-II** **Cartilage catabolism**	AC-08F1, Immunodiagnostic Systems, Dijon, France	3.7 pg/mL	Serum
**COMP** **Osteoarthritis**	MBS006795, MyBioSource, San Diego, USA	15.6 ng/mL	Serum
**HA** **Osteoarthritis**	TE1017-2, TECOmedical group, Eurobio Scientific, Les Ulis, France	2.7 ng/mL	Serum
**PGE_2_** **Inflammation**	KGE004B, R&D, Bio-Techne SAS, Noyal Châtillon sur Seiche, France	16 pg/mL	Heparin plasma
**IL-6** **Inflammation**	EHS0002, FineTest, Clinisciences, Nanterre, France	3.125 pg/ml	EDTA plasma

**Table 2:** Antibodies used for histological analyses.

Primary Antibody	Reference	Dilution	Secondary Antibody Reference	Dilution
**CYP17A1**	sc-374244 mouse monoclonal IgG, Santa Cruz Biotechnology, Dallas, TX, USA	1:50	7076S, anti-mouse IgG-HRP, Cell Signaling Technology, Leiden, The Netherlands	1:200
**Aromatase**	sc-374176 mouse monoclonal IgG, Santa Cruz Biotechnology, Dallas, TX, USA	1:50		
**ESR1**	GTX22746 mouse monoclonal IgG, Diagomics, Blagnac, France	1:50	sc-2005, goat anti-mouse IgG-HRP, Santa Cruz Biotechnology, Dallas, TX, USA	1:200
**AR**	sc-815 rabbit polyclonal IgG, Santa Cruz Biotechnology, Dallas, TX, USA	1:50	ab 205718, goat pAb anti IgG-HRP, Abcam, Paris, France	1:200

## Data Availability

The data are provided in the study.

## Supplementary Materials

The supporting information can be downloaded at: https://www.mdpi.com/article/10.3390/ijms242316778/s1.
