# Supplementary material for: Early Castration in Horses Does Not Impact Osteoarticular Metabolism
Source: Int J Mol Sci. 2023 Nov 26;24(23):16778. doi: 10.3390/ijms242316778 (PMC10706761; doi:10.3390/ijms242316778)
Supplement: Supplementary file 1 [file ijms-24-16778-s001.zip › ijms-2661823-supplementary.pdf]

Article

Early castration in horses does not impact osteoarticular metabolism

Marion Rouge [1,†], Florence Legendre [2,†], Razan Elkhatib [1], Christelle Delalande [1], Juliette Cognié [3], Fabrice Reigner [4], Philippe Barrière [4], Stefan Deleuze [5], Vincent Hanoux [1], Philippe Galéra [2], Hélène Bouraïma-Lelong [1,*]

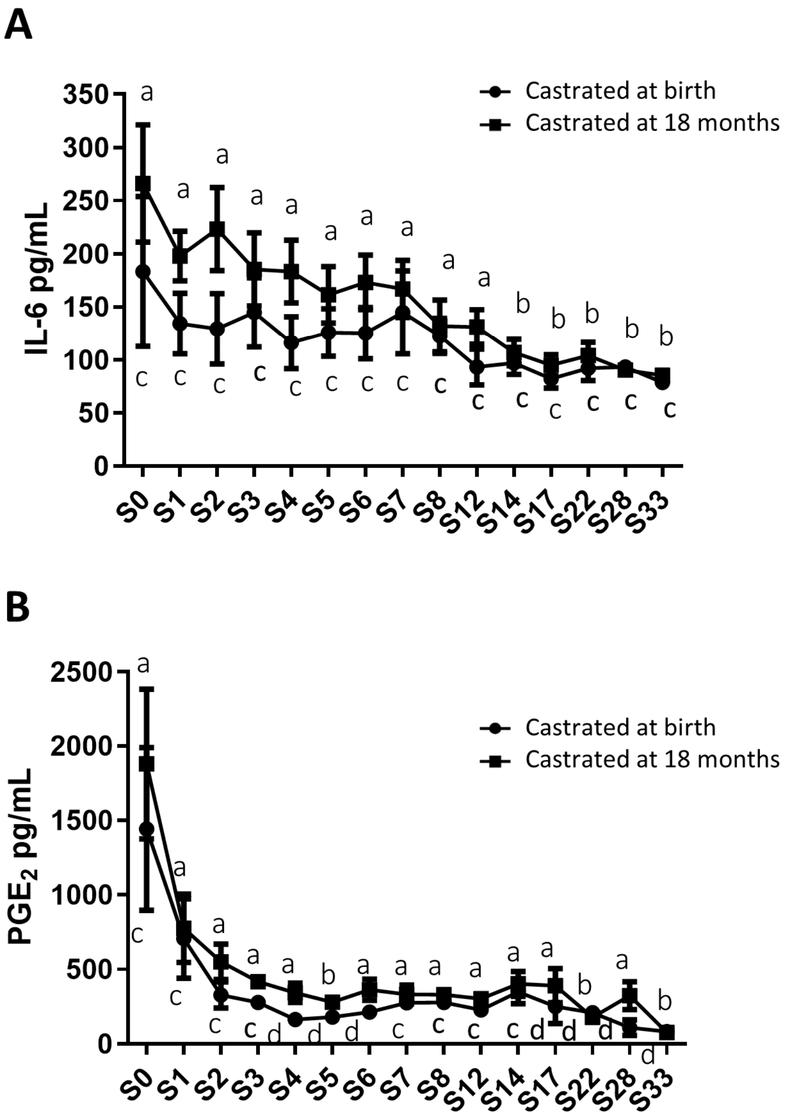

Figure S1. Circulating levels of inflammatory biomarkers in plasma samples from horses castrated at birth and at 18 months. Plasma levels of IL-6 (A) and PGE2 (B) were measured for 33 months after birth in the two experimental groups using ELISA (S0 to S12: n=11, S14 to S17: n=8, S28 to S33: n=5). Data are presented as mean ± SEM. A variation analysis in the biomarker levels compared to P0 within the same group was performed with a Kruskal-Wallis test followed by a Dunn's comparison (a ≠ b; c ≠ d). A Mann-Witney test was compared to compare the biomarker levels at the same time between the two groups, and no differences were observed.
